# TR score: A noninvasive model to predict histological stages in patients with primary biliary cholangitis

**DOI:** 10.3389/fimmu.2023.1152294

**Published:** 2023-03-16

**Authors:** Zhixin Tu, Yao Wang, Yan Wang, Jianjie Huang, Yujin Han, Qijia Ji, Xiaoxuan Cao, Xiaoyu Wen, Yang Wang, Qinglong Jin

**Affiliations:** ^1^ Department of Hepatology, The First Hospital of Jilin University, Changchun, Jilin, China; ^2^ Department of Gastroenterology, Qingdao Municipal Hospital, Qingdao, China

**Keywords:** primary biliary cholangitis, noninvasive model, total bile acid, red blood cell distribution width, histological stage

## Abstract

**Introduction:**

The aim of this study was to develop a noninvasive prediction model for histological stages in PBC that is simple, easy to implement, and highly accurate.

**Methods:**

A total of 114 patients with PBC were included in this study. Demographic, laboratory data and histological assessments were collected. The independent predictors of histological stages were selected to establish a noninvasive serological model. The scores of 22 noninvasive models were calculated and compared with the established model.

**Results:**

This study included 99 females (86.8%) and 15 males (13.2%). The number of patients in Scheuer’s stage 1, 2, 3 and 4 was 33 (29.0%), 34 (29.8%), 16 (14.0%), and 31 (27.2%), respectively. TBA and RDW are independent predictors of PBC histological stages. The above indexes were used to establish a noninvasive model-TR score. When predicting early histological change (S1) or liver fibrosis and cirrhosis (S3-S4), the AUROC of TR score were 0.887 (95% CI, 0.809-0.965) and 0.893 (95% CI, 0.816-0.969), higher than all of the other 22 models included in this study. When predicting cirrhosis (S4), its AUROC is still as high as 0.921 (95% CI, 0.837-1.000).

**Conclusion:**

TR score is an easy, cheap and stable noninvasive model, without complex calculation formulas and tools, and shows good accuracy in diagnosing the histological stages of PBC.

## 1 Introduction

Primary biliary cholangitis (PBC, formerly known as primary biliary cirrhosis), is a chronic autoimmune liver disease characterized by bile duct epithelial cell damage, cholestasis, and autoantibody production. In the last 50 years, the incidence of PBC has been on the rise. In addition to the improvement in diagnostic capabilities (detection of anti-mitochondrial antibodies, etc.) and the increased awareness of this disease, the incidence of PBC is still realistically increasing in regions such as the Asia-Pacific region, Italy, and the Netherlands, which may be related to the increase in the aging population and changes in environmental causative factors (1, 2). The pathogenesis of PBC remains poorly understood. It is currently believed that genetic factors, epigenetic and environmental triggers underlie the pathogenesis of PBC, the consequence of which is the combination of innate and acquired immune response to biliary tract injury and bile acid physiology (3–5).

The histology of PBC is characterized by chronic non-suppurative destructive cholangitis involving the interlobular and septal bile ducts, and the typical changes are severe inflammatory infiltrates and necrotic focal lesions around the bile ducts, called ‘florid duct lesions’ (6, 7). The pathological manifestations of PBC are usually divided into four stages: stage 1 is characterized by inflammation of the confluent area with or without bile duct lesions, in which characteristic florid duct lesions are seen; stage 2 is characterized by the spread of inflammation of the confluent area to the liver parenchyma, with fine bile duct hyperplasia and the formation of interfacial hepatitis; stage 3 is characterized by structural changes of the liver with visible fibrous septa; and stage 4 by the presence of cirrhosis with regenerative nodules (8, 9).

Due to the high specificity of serum markers, liver biopsy is not mandatory for the diagnosis of PBC. The European Association for the Study of the Liver (EASL) recommends monitoring the progression of PBC by vibration-controlled transient elastography (VCTE) (7). A previous study has shown that liver stiffness measurement (LSM) ≤6.5 and >11.0 kPa measured by VCTE can identify the presence of advanced fibrosis in patients with PBC (10). Whereas VCTE is not reliable for patients with LSM between these two cut-off values, and VCTE is weak for the identification of early lesions in PBC.

Therefore, T he use of noninvasive models to predict the prognosis and hepatic histological changes in patients with PBC is essential in clinical practice. Recent studies have shown that the albumin-bilirubin score (ALBI) reflects the pathological staging of Japanese PBC patients and that a lower ALBI score before ursodeoxycholic acid (UDCA) prescription predicts better liver transplant-free survival (11). Elevated aspartate aminotransferase (AST) to platelet ratio index (APRI) has also been validated in different cohorts to be associated with the risk of adverse events in patients with PBC (12). A study that included 35 patients with biopsy-proven PBC showed that the gamma-glutamyl transferase (GGT) to platelet ratio (GPR) was more sensitive than APRI and FIB-4 (fibrosis index based on the four factors) in detecting advanced fibrosis in patients with PBC (13). A Korean study showed that the neutrophil-to-lymphocyte ratio (NLR) was significantly associated with transplant-free survival in patients with PBC (14). The red blood cell distribution width (RDW) to platelet ratio (RPR) can be used as a predictor of significant liver fibrosis and cirrhosis in patients with chronic hepatitis B. It has been shown that RPR can provide useful information for predicting the histological severity of PBC (15, 16). AST to alanine aminotransferase (ALT) ratio (AAR) was significantly different between the presence or absence of cirrhosis in chronic liver disease, but its correlation with the liver fibrosis stage in PBC was poor (17, 18).

In addition, noninvasive models commonly used in clinical to predict the prognosis and severity of fibrosis include age-platelets simplifies index (AP index), three-parameter cirrhosis discriminant score (CDS), Doha score, fibrosis cirrhosis index (FCI), fibrosis index (FI), FIB-4, fibro-quotient (FibroQ), globulin/platelet model (GP model), Göteburg University Cirrhosis Index (GUCI), hepatitis B-fibrosis score (HB-F), Lok index, King’s score, non-invasive Koelin-Essen-index (NIKEI), Pohl model, S index, and model of end-stage liver disease(MELD) (19–33). However, these noninvasive models are mostly used for other types of chronic liver diseases (such as viral and fatty liver diseases). Noninvasive models applied to PBC in the clinic are still relatively few, and there is a lack of simple, inexpensive, and easy-to-use scoring models. This study aims to find a noninvasive prediction model for histological lesions in PBC that is simple, easy to implement, and highly accurate.

## 2 Method

### 2.1 Patient and laboratory testing

A total of 114 patients with PBC who underwent liver biopsy from January 1^st^, 2013 to October 31, 2022, at the First Hospital of Jilin University were enrolled in this study. Inclusion criteria include: (1) meeting the diagnostic criteria for PBC published by the American Association for the Study of Liver Diseases(AASLD) in 2018 (6); (2) being either untreated with UDCA or non-responders to UDCA treatment. Demographic and laboratory data within the last week before the liver biopsy were collected. Exclusion criteria included: (1) combined with other types of liver disease, such as viral hepatitis, autoimmune hepatitis, drug-related liver disease, alcoholic liver disease or non-alcoholic fatty liver disease; (2) combined with severe cardiovascular disease; (3) combined with malignant tumor or other important organ failures; (4) incomplete information on clinical data. Laboratory data include AST, ALT, GGT, alkaline phosphatase (ALP), albumin (ALB), globulin (GLB), total bilirubin (TBil), total bile acid (TBA), neutrophil count (NE), lymphocyte count (LY), RDW, platelet count(PLT), international normalized ratio(INR), prothrombin time(PT) and serum creatinine(SCr).

### 2.2 Formulas

The formulas of the 23 noninvasive models included in this study are shown in the **Supplementary Material**.

### 2.3 Histological assessment

After obtaining the patient’s informed consent, a liver biopsy was performed under color Doppler ultrasound guidance with 18G Tru-Cut needles. The puncture required a liver tissue length of 1 to 2.2 cm, including more than 6 intact confluent areas. The specimens were fixed with 10% formaldehyde solution, embedded in paraffin, and stained with hematoxylin and eosin(H&E) and argentophilic staining methods. The pathological diagnosis of each liver tissue was determined after a double-blind examination by 2 experts from the Pathological Diagnostic Center of the First Hospital of Jilin University, and histological staging was performed using Scheuer’s staging method.

### 2.4 Statistical methods

SPSS 25.0 and Graghpad Prism 9.0 software were applied for statistical analysis and graphing of data. The quantitative data were expressed as mean ± standard deviation or median (interquartile range), and intergroup comparisons were analyzed by independent samples t-test or Mann-Whitney nonparametric test. The qualitative or categorical data were expressed as percentages, and the intergroup comparisons were measured by the chi-square test. One-way ANOVA or K independent samples nonparametric test was applied to analyze the predictors associated with the histological stages of PBC. Grade correlation analysis was performed using Spearman correlation analysis. Independent predictors associated with histological stages were analyzed using multivariate ordered logistic regression, and the selected independent variables were modeled by multivariate stepwise regression analysis. The receiver operating curve (ROC) was applied to assess the predictive value of each noninvasive model. The best cutoff points were selected according to the Youden index from the ROC curve. The accuracy of the constructed model was evaluated by consistency, sensitivity, specificity, positive predictive value (PPV), negative predictive value (NPV) and consistency test with pathological diagnosis. P values< 0.05 was considered statistically significant.

## 3 Result

This study included 114 PBC patients, including 99 females (86.8%) and 15 males (13.2%). The ratio of male vs. female was 1:6.6, and the median age was 53 (48,58) years. The number of patients in Scheuer’s stage 1, 2, 3 and 4 was 33 (29.0%), 34 (29.8%), 16 (14.0%), and 31 (27.2%), respectively. All patients were randomly divided into a model group (n=78) and a validation group (n=36). The general information of the two groups is shown in **Table 1**. There was no statistically significant difference between the model group and the validation group.

**Table 1:** General information of model group and validation group.

General information	Model group (n=78)	Validation group (n=36)	P value
Demographic			
Age (yr)	52.78 ± 8.51	52.50 ± 8.27	0.868
Female (%)	69 (88.4%)	30 (83.3%)	0.451
Biochemical			
AST (U/L)	73.5 (40.2, 113.85)	61.3 (43.025, 98.7)	0.692
ALT (U/L)	64.9 (35.45, 114)	46.85 (34.75, 93.53)	0.271
GGT (U/L)	261 (82.05, 524)	234.8 (85.18, 451.25)	0.448
ALP (U/L)	236.6 (155.2, 405.5)	195.15 (138.08, 337.75)	0.195
ALB (g/L)	35.95 ± 6.17	35.56 ± 4.40	0.699
GLB (g/L)	35.00 ± 6.44	35.68 ± 6.58	0.603
TBil (umol/L)	26.9 (14.15, 81.6)	16.6 (11, 99.53)	0.274
TBA (umol/L)	36.2 (11.1, 141.3)	41.3 (12.1, 89.7)	0.953
NE (10^9^/L)	2.46 (2.05, 3.14)	2.555 (2.13, 3.07)	0.878
LY (10^9^/L)	1.52 (0.9, 1.87)	1.54 (0.97, 2.025)	0.458
RDW (%)	14.4 (13.4, 16.6)	14.25 (13.48, 17.1)	0.882
PLT (10^9^/L)	157 (100, 225)	181.5 (141.75, 237.5)	0.215
PT (s)	11.1 (10.5, 12.45)	11.2 (10.55, 13.7)	0.919
INR	0.97 (0.9, 1.10)	0.975 (0.91. 1.17)	0.6
SCr (umol/L)	52 (46.45, 61.85)	57.95 (51.28, 63.98)	0.093
Histological stage			
S1	22 (28.2%)	11 (30.6%)	
S2	21 (26.9%)	13 (36.1%)	0.561
S3	13 (16.7%)	3 (8.3%)	
S4	22 (28.2%)	9 (25%)	

**Figure 1:**
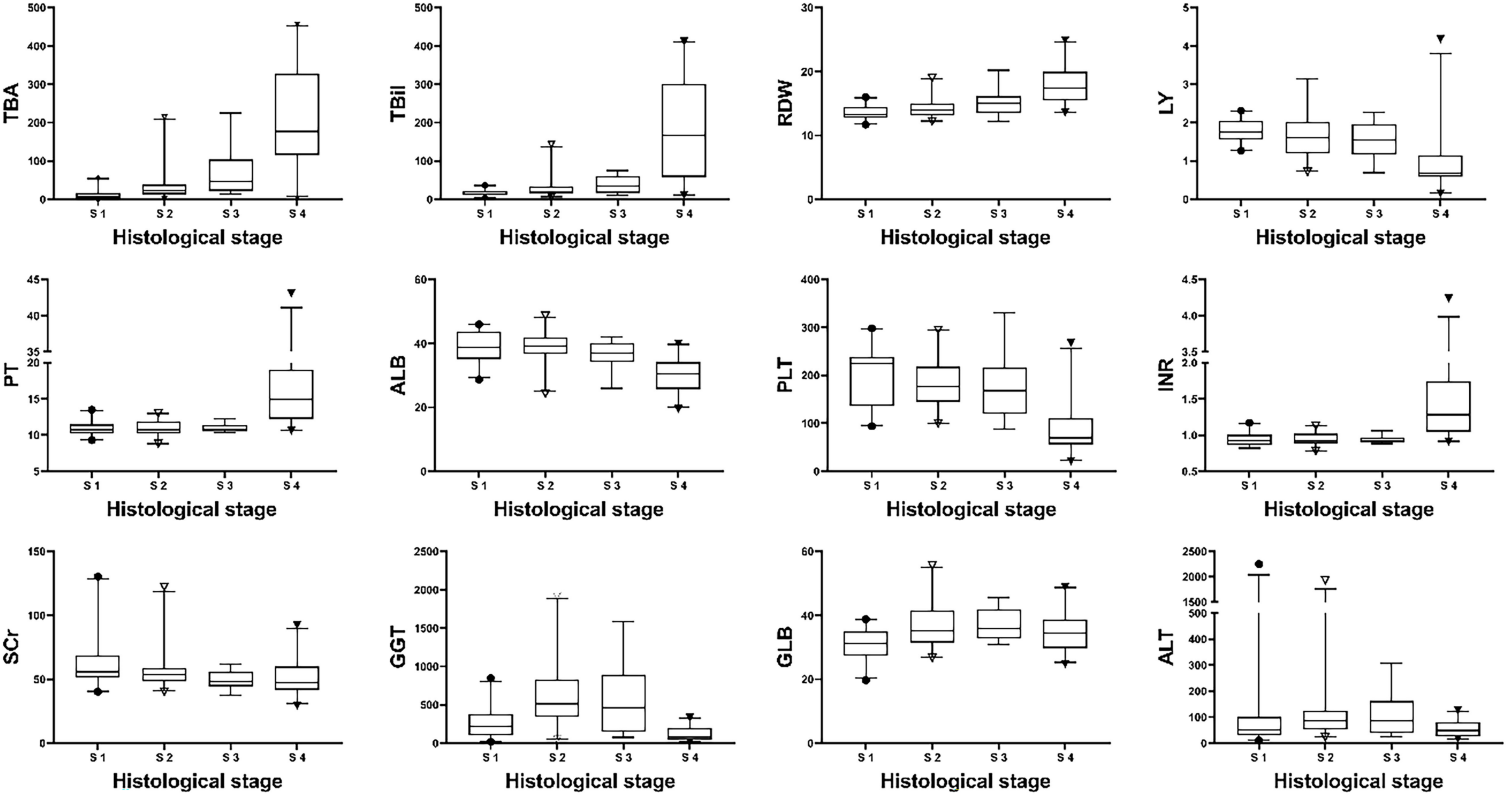
Distribution of biochemical parameters with histological stages in the model group. Note: The top and bottom of each box are the 25th and 75th percentiles, the horizontal line through the box is the median, and the error bars are the 5% and 95% percentiles.

The clinical characteristics of patients with different pathological stages in the model group are shown in **Table 2**. All indicators except age, sex, AST and NE differed among different pathological stages (P<0.05). Bivariate Spearman correlation analysis showed that TBA, TBil, RDW, LY, PT, ALB, PLT, INR, SCr, GGT, AST, and GLB were significantly correlated with pathological stage grade (**Table 3**). The block diagrams of TBA, TBil, RDW, LY, PT, ALB, PLT, INR, SCr, GGT GLB and ALT according to the histological stage were shown in **Figure 1**. It can be seen that with the progression of the histological stage, the levels of TBA, TBil and RDW gradually increased, while the levels of LY, ALB and PLT decreased. PT, INR, SCr, GGT, GLB and ALT changed less significantly with histological stages.

**Table 2:** Clinical characteristics of patients with different histological stages in the model group.

Clinical characteristics	S1 (n=22)	S2 (n=21)	S3 (n=13)	S4 (n=22)	P value
Age (yr)	52.5 ± 9.32	53.24 ± 9.67	49.92 ± 5.59	54.32 ± 7.98	0.526
Female (%)	19 (86.4%)	17 (81.0%)	13 (100%)	20 (91%)	0.379^a^
AST (U/L)	32.30 (24.00, 93.00)	81.25 (61.50, 123.58)	100.85 (40.63, 126.80)	73.05 (54.55, 115.85)	0.091
ALT (U/L)	47.80 (28.50, 120.00)	86.70 (56.18, 130.20)	99.50 (41.55, 166.45)	48.35 (25.83, 78.90)	0.031
GGT (U/L)	198.00 (99.60, 357.00)	524.00 (344.18, 853.75)	521.85 (188.95, 941)	77.65 (39.45, 192.28)	<0.001
ALP (U/L)	163.00 (96.20, 242.70)	383.50 (231.478, 461.10)	300.65 (181.55, 580.53)	213.25 (147.33, 280.20)	0.013
ALB (g/L)	39.02 ± 5.04	38.72 ± 4.79	35.90 ± 5.09	30.25 ± 5.10	<0.001
GLB (g/L)	31.25 ± 4.64	36.98 ± 7.35	37.12 ± 4.75	35.45 ± 6.64	0.011
TBil (umol/L)	13.40 (10.50, 15.50)	21.40 (15.40, 36.08)	34.95 (16.18, 60.58)	166.40 (57.98, 300.68)	<0.001
TBA (umol/L)	6.40 (3.40, 13.90)	23.65 (15.43, 38.63)	46.55 (21,95, 104.10)	177.10 (115.00, 327.08)	<0.001
NE (10^9^/L)	2.68 (2.22, 3.93)	2.67 (2.03, 3.12)	2.51 (2.06, 3.03)	2.28 (1.49, 2.84)	0.494
LY (10^9^/L)	1.76 (1.62, 2.08)	1.61 (1.20, 2.02)	1.40 (1.17, 1.89)	0.69 (0.59, 1.14)	<0.001
RDW (%)	13.20 (12.80, 14.40)	13.95 (13.15, 14.95)	14.65 (13.53, 16.43)	17.35 (15.45, 19.95)	<0.001
PLT (10^9^/L)	228.00 (147.00, 241.00)	176.00 (144.75, 218.25)	167.00 (119.50, 195.25)	69.50 (56.25, 100.25)	<0.001
PT (s)	10.60 (10.30, 11.40)	10.70 (10.25, 11.83)	10.70 (10.45, 11.18)	14.90 (12.23, 19.03)	<0.001
INR	0.92 (0.86, 1.00)	0.92 (0.88, 1.02)	0.92 (0.90, 0.96)	1.28 (1.05, 1.74)	<0.001
SCr (umol/L)	55.90 (51.50, 68.80)	53.60 (47.75, 59.08)	47.45 (43.63, 55.15)	47.00 (41.63, 59.90)	0.019

The meaning of a is the chi-square value.

**Table 3:** Correlation analysis between clinical characteristics and histological stages in the model group.

Clinical characteristics	R value	P value
TBA	0.746	<0.0001
TBil	0.675	<0.0001
RDW	0.638	<0.0001
LY	-0.586	<0.0001
PT	0.563	<0.0001
ALB	-0.550	<0.0001
PLT	-0.549	<0.0001
INR	0.544	<0.0001
SCr	-0.348	0.002
GGT	-0.280	0.013
AST	0.229	0.044
GLB	0.227	0.047
NE	-0.160	0.165
ALT	-0.116	0.313
sex (Female=1, male=2)	-0.100	0.385
ALP	0.068	0.557
age	0.005	0.969

All eight serological parameters in **Table 3** with correlation coefficient >0.5 in absolute value were included in the multivariate ordered logistic regression analysis. It was suggested that only TBA (adjusted OR: 1.017,95%CI, 1.004-1.030) and RDW (adjusted OR: 1.647, 95%CI, 1.114-2.514) were independent predictors of PBC histological stage (p<0.05). Multiple stepwise regression analysis of TBA and RDW with PBC histologic stage was performed. The adjusted R-squared after incorporation both TBA and RDW was 0.563, and the regression model was:


Model S=−0.668+0.005×TBA+0.176×RDW

To facilitate calculation and application in the clinic practice, the model was simplified to:


TR score(TBA−RDW score)=TBA×RDW/10

The validity of TR score in predicting the histological stages of PBC will be compared with Model S and 22 other models in the following.

TR scores, Model S and other 22 noninvasive models were calculated for patients in the model group according to the formula shown in the **Supplementary Material**. 24 models were analyzed for Spearman correlation with the pathological stage as shown in **Table 4**. All models except the S index and GPR showed significant correlation (p<0.05) with the pathological stage of PBC, with the TR score showing the strongest correlation (r=0.770, p<0.0001). The correlation of TR score is similar to Model S.

**Table 4:** Correlation analysis between 24 models and the histological stage of PBC in the model group.

Noninvasive models	R value	P value
TR score	0.770	<0.0001
Model S	0.761	<0.0001
FCI	0.719	<0.0001
ALBI	0.658	<0.0001
RPR	0.656	<0.0001
MELD	0.652	<0.0001
FIB-4	0.635	<0.0001
FI	0.623	<0.0001
GP model	0.617	<0.0001
Lok index	0.610	<0.0001
FibroQ	0.604	<0.0001
CDS	0.603	<0.0001
NIKEI	0.591	<0.0001
HB-F	0.590	<0.0001
GUCI	0.568	<0.0001
King's score	0.553	<0.0001
Doha score	0.552	<0.0001
AAR	0.518	<0.0001
APRI	0.503	<0.0001
NLR	0.454	<0.0001
Pohl model	0.413	<0.0001
AP index	0.380	<0.001
S index	0.037	0.749
GPR	-0.033	0.779

**Figure 2:**
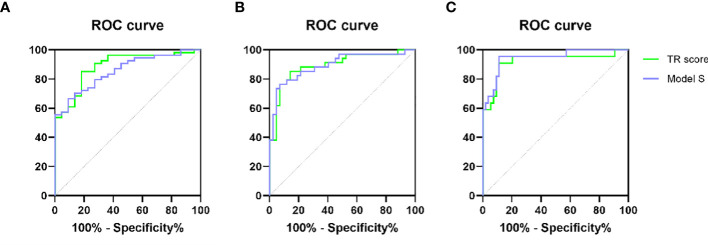
ROC generated by TR score and Model S for prediction of S2-S4 **(A)**, S3-S4 **(B)** and S4 **(C)**.

The patients in the model group were divided into stage 1 (S1) and stage 2-4 (S2-4) according to pathological stages. The receiver operating curve (ROC) of the TR score and Model S for predicting the histological stage of S1 and S2-S4 was shown in **Figure 2A**. The AUROC, sensitivity, specificity and cutoff value of 23 models were shown in **Table 5**. The AUROC of TR score, Model S and FCI was more than 0.85, which were considered to be effective in predicting PBC with histological stage S2-4. The AUROC of TR score was 0.887 (95% CI, 0.809~0.965), which was higher than all other noninvasive models included in the study, indicating that its ability to distinguish histological changes in ultra-early stage (S1) was better than that of the other 23 models. The cutoff value of the TR score for prediction S2-S4 is 23.923, and the sensitivity, specificity, PPV and NPV of the TR score is 85.2%, 81.8%, 92.0%, 69.2%, respectively.

**Table 5:** Validity of each models for prediction of S2-S4.

Noninvasive model	AUROC (95%CI)	Sensitivity(%)	Specificity (%)	Cutoff value	P value
TR score	0.887 (0.809, 0.965)	85.2	81.8	>23.923	<0.0001
Model S	0.854 (0.771, 0.938)	66.7	90.9	>2.044	<0.0001
FCI	0.856 (0.764, 0.948)	83.3	71.4	>0.345	<0.0001
FIB-4	0.800 (0.682, 0.918)	85.5	68.2	>1.700	<0.0001
GP model	0.796 (0.687, 0.905)	83.6	66.7	>1.725	<0.0001
NIKEI	0.794 (0.677, 0.910)	74.6	81.0	>0.855	<0.0001
MELD	0.784 (0.679, 0.888)	53.7	94.7	>9.5	<0.0001
GUCI	0.780 (0.656, 0.904)	76.4	72.7	>1.005	<0.001
RPR	0.773 (0.663, 0.882)	83.6	59.1	>0.065	<0.001
ALBI	0.771 (0.661, 0.881)	47.3	95.2	>-1.945	<0.001
King's score	0.767 (0.642, 0.892)	74.6	77.3	>20.35	<0.001
APRI	0.765 (0.631, 0.898)	89.1	63.6	>0.670	<0.001
Doha score	0.758 (0.643, 0.872)	83.6	63.6	>4.335	<0.001
FI	0.743 (0.630, 0.856)	63.6	77.3	>2.560	<0.001
FibroQ	0.729 (0.608, 0.849)	76.4	63.6	>2.280	<0.01
Lok index	0.727 (0.610, 0.843)	41.8	95.5	>0.565	<0.01
CDS	0.726 (0.607, 0.845)	74.5	63.6	>4.5	<0.01
HB-F	0.725 (0.605, 0.845)	76.4	59.1	>1.185	<0.01
AAR	0.677 (0.546, 0.807)	53.6	77.3	>1.195	<0.05
NLR	0.670 (0.541, 0.800)	81.5	50.0	>1.400	<0.05
S index	0.668 (0.536, 0.800)	76.4	57.1	>0.855	<0.05
GPR	0.655 (0.524, 0.785)	56.4	72.7	>3.530	<0.05
AP index	0.664 (0.510, 0.818)	74.5	68.2	>4.500	<0.05
Pohl model	0.573 (0.433, 0.712)	0.418	72.7	Positive	>0.05

Patients in the model group were divided into stage 1-2 (S1-S2) and stage 3-4 (S3-S4). The ROC of the TR score and Model S for predicting the histological stage of S3-S4 was shown in **Figure 2B**. AUROC, sensitivity, specificity and cutoff value of 24 models were shown in **Table 6**. The AUROC of TR score, Model S, ALBI and FCI were more than 0.85, indicating that the above 4 models were effective in predicting PBC at S3-S4. The AUROC of TR score for prediction S3-S4 in patients with PBC was 0.893 (95%CI, 0.816-0.969), which was higher than that of the other 22 existing models, indicating that its ability to differentiate fibrosis and cirrhosis (S3-S4) was better than that of all other models included in this study. The cutoff value of the TR score for prediction S3-S4 is 61.189, and the sensitivity, specificity, PPV and NPV of the TR score is 85.3%, 85.7%, 82.9%, 87.8%, respectively.

**Table 6:** Validity of each models for prediction of fibrosis and cirrhosis (S3-S4).

Noninvasive model	AUROC (95%CI)	Sensitivity(%)	Specificity (%)	Cutoff value	P value
TR score	0.893 (0.816,0.969)	85.3	85.7	>61.189	<0.0001
Model S	0.894 (0.818,0.970)	79.4	88.1	>2.231	<0.0001
ALBI	0.859 (0.774,0.944)	70.6	92.9	>-1.945	<0.0001
FCI	0.858 (0.773,0.942)	64.7	97.6	>2.24	<0.0001
RPR	0.827 (0.734,0.921)	68.6	83.3	>0.115	<0.0001
FI	0.825 (0.731, 0.919)	74.3	78.6	>2.85	<0.0001
MELD	0.819 (0.717, 0.920)	70.6	84.6	>9.5	<0.0001
CDS	0.796 (0.696,0.897)	42.9	1	>7.5	<0.0001
GP model	0.795 (0.690, 0.901)	60.0	92.7	>3.22	<0.0001
Lok index	0.794 (0.691,0.897)	60.0	92.9	>0.565	<0.0001
FIB-4	0.791 (0.691, 0.890)	82.9	64.3	>2.415	<0.0001
FibroQ	0.789 (0.687, 0 0.892)	65.7	83.3	>4.705	<0.0001
HB-F	0.785 (0.680,0.889)	65.7	85.7	>1.13	<0.0001
NIKEI	0.763 (0.654,0.871)	73.5	73.8	>0.995	<0.0001
Doha score	0.759 (0.647, 0.871)	74.3	71.4	>5.435	<0.0001
GUCI	0.759 (0.652,0.866)	77.1	69.1	>1.325	<0.0001
AAR	0.754 (0.646,0.861	68.6	74.4	>1.195	<0.0001
King's score	0.750 (0.641, 0.859)	68.6	78.6	>35.165	<0.001
NLR	0.741 (0.629,0.85	61.8	78.6	>2.095	<0.001
APRI	0.727 (0.614,0.839)	71.4	69.0	>1.525	<0.001
Pohl model	0.679 (0.556,0.801)	57.1	78.6	Positive	<0.01
AP index	0.661 (0.534,0.787)	85.7	57.1	>4.5	<0.05
GPR	0.557 (0.428, 0.687)	68.6	47.6	<4.395	>0.05
S index	0.511 (0.380, 0.643)	74.3	36.6	<2.205	>0.05

Patients in the model group were divided into stages 1-3 (S1-S3) and stage 4 (S4) according to pathological stages. The ROC of the TR score and Model S for predicting S4 was shown in **Figure 2C**, and the AUROC, sensitivity, specificity and cutoff value of each model were shown in **Table 7**. Many models showed well performance for the prediction of S4, with the AUROC of TR score, Model S, Lok index, FibroQ, RPR, HB-F, CDS, FI, FCI, ALBI and MELD over 0.9. The AUROC of TR score for prediction of cirrhosis (S4) in PBC was 0.921 (95%CI, 0.837-1.000), which had great predictive ability and ranked 7th among 24 models. The cutoff value of the TR score for predicting S4 is 97.465, and the sensitivity, specificity, PPV and NPV of the TR score is 90.9%, 88.9%, 76.9%, 96.0%, respectively.

**Table 7:** Validity of each models for prediction of cirrhosis (S4).

Noninvasive model	AUROC (95%CI)	Sensitivity(%)	Specificity (%)	Cutoff value	Pvalue
TR score	0.921 (0.837,1.000)	90.9	88.9	>97.465	<0.0001
Model S	0.944 (0.887,1.000)	90.9	88.9	>2.401	<0.0001
Lok index	0.949 (0.894,1.000)	86.4	92.7	>0.575	<0.0001
FibroQ	0.941 (0.887,0.995)	90.9	83.7	>4.81	<0.0001
RPR	0.929 (0.860,0.999)	86.4	89.1	>0.135	<0.0001
HB-F	0.926 (0.858,0.995)	90.9	87.3	>1.250	<0.0001
CDS	0.925 (0.855,0.995)	77.3	90.9	>6.5	<0.0001
FI	0.916 (0.852,0.980)	77.3	92.7	>3.435	<0.0001
FCI	0.910 (0.831,0.990)	81.8	92.5	>2.280	<0.0001
FIB-4	0.907 (0.838,0.977	77.3	90.9	>5.605	<0.0001
ALBI	0.904 (0.831,0.976)	77.3	92.6	>-1.59	<0.0001
MELD	0.903 (0.812,0.994)	77.3	98.0	>12.5	<0.0001
AAR	0.898 (0.825,0.971)	86.4	78.6	>1.245	<0.0001
GP model	0.875 (0.764,0.985)	77.3	94.4	>3.815	<0.0001
GUCI	0.852 (0.763,0.941)	90.9	63.6	>1.325	<0.0001
King's score	0.852 (0.765,0.940)	81.8	72.7	>35.165	<0.0001
Doha score	0.850 (0.747,0.954)	77.3	83.6	>.6.595	<0.0001
NIKEI	0.815 (0.719,0.911)	90.9	70.4	>0.995	<0.0001
NLR	0.810 (0.691,0.929)	61.9	94.6	>2.830	<0.0001
Pohl model	0.809 (0.697,0.921)	81.8	80.0	Positive	<0.0001
APRI	0.794 (0.691,0.896	81.8	65.5	>1.625	<0.0001
AP index	0.770 (0.668,0.872)	90.9	56.4	>5.5	<0.0001
GPR	0.676 (0.551,0.802)	63.6	70.9	<2.760	<0.05
S index	0.615 (0.482,0.747)	81.8	46.3	<1.560	>0.05

In predicting S2-S4 (0.887 and 0.854), S3-S4 (0.893 and 0.894), and S4 (0.921 and 0.944), the AUC values of TR score and Model S were very close, and the sensitivity and specificity were close. In terms of calculation, TR score is simpler than Model S, and the diagnostic criteria of Model S are very close, while which of TR model span more, therefore, TR score is chosen as the final prediction model.

The ability of the TR score to predict the histological stage was analyzed in 36 patients in the validation group. TR score>23.923 was used as the diagnostic criteria for distinguishing histological manifestations of S2-S4, and the accuracy, sensitivity, specificity, PPV and NPV of the TR score were 72.2%, 80%, 54.5%, 83.3% and 54.5%, respectively. After the consistency test, the kappa value of the pathological gold standard was 0.345 (P<0.05). Using TR score>61.189 as the diagnostic criteria for distinguishing S3-S4, the consistency rate, sensitivity, specificity, specificity, PPV and NPV were 75.0%, 83.3%, 70.8%, 58.8% and 89.5%, respectively. The kappa was 0.491 (P<0.01). Using TR score>97.465 as the diagnostic criteria for distinguishing S4, the consistency rate, sensitivity, specificity, specificity, PPV and NPV were 80.6%, 77.8%, 81.5%,58.3% and 91.7%, respectively. The kappa was 0.533 (P<0.01).

## 4 Discussion

PBC is a chronic cholestatic disease characterized by the destruction of intrahepatic bile ducts, leading to fibrosis and potential cirrhosis (34). Because of the high specificity of immunological markers, liver biopsy is not necessary for the diagnosis of PBC. However, when PBC-specific antibodies are negative or autoimmune hepatitis or nonalcoholic fatty liver disease is suspected, liver biopsy is still necessary (7). Advanced histological stages are closely related to poor curative effect and prognosis in patients with PBC (35–37). For patients with inadequate response to UDCA, dynamic monitoring of liver histological lesions is helpful to adjust the treatment plan in time (7). However, sampling errors, contraindications, poor compliance and economic conditions and complications including bleeding and death limit the implementation of liver biopsy (38). Most of the noninvasive models in clinical are used for the evaluation of viral or fatty liver disease, but few models are used in PBC, and most of the models are complex in the calculation, which is not convenient for clinical application. The aim of this study is to find a simple, easy and accurate noninvasive model for predicting the histological stages in PBC.

A total of 114 patients with PBC were included in this study, with a ratio of male to female of 1: 6.6. Compared with the previous understanding that the ratio was about 1:9, this result is closer to the recent epidemiological findings (1:5-1:6) (39, 40). This study shows that TBA and RDW are independent predictors of PBC histological stages and are helpful to distinguish patients with different stages. The imbalance between bile acid synthesis and metabolism in PBC leads to excessive accumulation of cytotoxic bile acids. Hydrophobic bile acid inhibits the expression of anion exchange protein 2 (AE2) by inducing oxidative stress and increases the expression of immune-related cell surface markers HLA-DR and CD40 on biliary epithelial cells (BECs); Down-regulation of AE2 activates soluble adenylyl cyclase, which makes BECs sensitive to apoptosis induced by bile salt (4, 41). RDW is measure of the variability of circulating red blood size and has previously played an important role in the differential diagnosis of anemia, with levels often increasing following ineffective red blood cell production (e.g. iron, vitamin B12 or folic acid deficiency), increased red blood cell destruction (e.g. hemolytic anemia) or after transfusion therapy (42). Elevated RDW levels are thought to be associated with acute and chronic heart failure, coronary artery disease, peripheral artery disease, end-stage renal disease, pulmonary hypertension, stroke, gram-negative bacteremia, alcoholic or non-alcoholic fatty liver disease, hepatitis B and all-cause mortality in adults (42–50). Elevated RDW levels in patients with liver disease may be associated with inflammatory states, impaired renal function, nutritional deficiencies, and increased erythrocyte destruction due to hypersplenism (48). This study also pointed out that there was no significant correlation between PBC histological stage with age, sex and neutrophil count. When the histological lesion progressed to S4, namely cirrhosis, the levels of AST, ALT, GGT and ALP decreased compared with those in the early stage.

To facilitate clinical calculation and application, a new noninvasive model was constructed by using the above two variables:


TR score=TBA×RDW/10

This study shows that the TR score is excellent in predicting the histological stages of PBC. When predicting early histological lesions (S1), the AUROC of TR score was 0.887 (95% CI, 0.809-0.965), which was higher than all of the other 22 models included in this study. In other models, FCI performed well in predicting the early stages of PBC, while the Pohl model had no significance. When predicting liver fibrosis and cirrhosis (S3-S4), the AUROC of TR score was 0.893 (95% CI, 0.816-0.969), which was also higher than that of the other 22 models. Among the other 22 models, ALBI also had a good predictive ability in addition to FCI, which supported the previous study that ALBI had a good prediction of the prognosis of PBC patients (11). When predicting cirrhosis (S4), most models have good prediction ability. Although the TR score does not show the best prediction ability compared with other models, its AUROC is still as high as 0.921 (95% CI, 0.837-1.000). It shows that the TR score has a great ability to predict the histology stages of PBC.

We analyzed the effectiveness of the TR score in another PBC cohort in the same center. When TR score ≤ 23.923 was used to predict early lesions, the specificity of the TR score was 80%, and the kappa value of the pathological gold standard was 0.345. When TR score > 61.189 was used to predict hepatic fibrosis and cirrhosis, the sensitivity (83.3%) and specificity (70.8%) of the TR score were higher, and the kappa value was 0.491. When TR score > 97.465 was used to predict liver cirrhosis, the sensitivity and specificity were 77.8% and 81.5%, and the kappa value was 0.533. In the validation group, although the TR score has a good ability to predict each histological stage, it is the most effective in predicting liver cirrhosis.

This study still has some limitations. This study is a retrospective study of a single center, so the included cases mainly represent the general manifestations of PBC patients in Northeast China. Further validation of the TR score at other centers is needed. More large-scale studies are needed to further refine the diagnostic criteria of the model and evaluate its ability to predict the prognosis of patients with PBC.

In summary, TBA and RDW are independent risk factors for advanced histological stages in PBC, and TR score has a great ability to predict the stage of histological. Compared with 22 existing noninvasive models, the TR score showed the highest AUROC in predicting early stage (S1) and hepatic fibrosis or cirrhosis (S3-S4), and excellent predictive ability in predicting cirrhosis (S4). The indexes included in the TRscore are easy to obtain, cheap and stable, without complex calculation formulas and tools, and show good accuracy in diagnosing the histological stages.

## Data availability statement

The original contributions presented in the study are included in the article/**Supplementary Material**. Further inquiries can be directed to the corresponding author.

## Ethics statement

The studies involving human participants were reviewed and approved by the Ethics Committee of the First Hospital of Jilin University. The patients/participants provided their written informed consent to participate in this study.

## Author contributions

Conceptualization, QJin and XW; methodology, ZT and QJin; formal analysis, ZT, JH and YanW; data curation, YaoW, QJi, XC and YH; writing—original draft preparation, ZT; writing-review and editing, YangW, XW and QJin; supervision, QJin; funding acquisition, QJin All authors have read and agreed to the published version of the manuscript.
