# Supplementary material for: TR score: A noninvasive model to predict histological stages in patients with primary biliary cholangitis
Source: Front Immunol. 2023 Mar 16;14:1152294. doi: 10.3389/fimmu.2023.1152294 (PMC10060872; doi:10.3389/fimmu.2023.1152294)
Supplement: Supplementary file 1 [file Table_1.docx]

| **Noninvasive Model** | | **Calculations** |
| --- | --- | --- |
| AAR | AST to ALT ratio | AST / ALT |
| ALBI | albumin-bilirubin score | log_10_ TBil × 0.66 + ALB × (-0.085) |
| AP index | age-platelets simplifies  index | AP index =age + PLT  age: <30=0, 30~39=1, 40~49=2, 50~59=3, 60~69=4,  ≥70=5  PLT: ≥225=0, 200~224=1, 175~199=2,150~174=3,  125~149=4, <125=5 |
| APRI | AST to PLT ratio index | (AST / ULN) × 100 / PLT |
| CDS | three-parameter cirrhosis  discriminant score | CDS = PLT + ALT/AST + INR  PLT: ≥340=0, 280~339=1, 220~279=2, 160~219=3, 100~159=4, 40~99=5, <40=6  ALT/AST: >1.7=0, 1.2~1.7=1, 0.6~1.19=2, <0.6=3  INR: <1.1=0, 1.1~1.4=1, >1.4 = 2 |
| Doha score |  | 8.5﹣0.2 × ALB + 0.01 × AST﹣0.02 × PLT |
| FCI | fibrosis cirrhosis index | (ALP × TBil) / (ALB × PLT) |
| FI | fibrosis index | 8.0﹣0.01 × PLT﹣ALB |
| FIB-4 | fibrosis index based on  the four factors | Age × AST / (PLT × ALT^1/2^) |
| FibroQ | fibro-quotient | 10 × age × AST × INR / (PLT × ALT) |
| GPR | GGT to PLT ratio | (GGT / ULN) × 100 / PLT |
| GP model | globulin/platelet model | GLB × 100 / PLT |
| GUCI | Göteburg University  Cirrhosis Index | (AST / ULN) × INR × 100 / PLT |
| HB-F | hepatitis B-fibrosis score | 0.018 × age + 1.085 × AST / ALT - 0.009 × PLT  + 0.449 × PT prolongation |
| King’s score |  | age × AST × INR / PLT |
| Lok index |  | Log odds =﹣5.56﹣0.0089 × PLT + 1.26 × AST/ALT  + 5.27 × INR  Predicted probability = exp(log odds) / [1+exp(log odds)] |
| MELD | model for end-stage liver  disease | 3.78 × ln TBil + 11.20 × ln INR + 9.57 × ln SCr + 6.43 |
| NIKEI | Noninvasive Koelin-  Essen-index | LogitP = ln(P/1-P)  =﹣24.214+0.225 × age + 0.056 × AST  +5.044 × AST/ALT + 3.631 × TBil |
| NLR | neutrophil-to-lymphocyte  ratio | NE / LY |
| Pohl model |  | Positive: AST/ALT≥1 and PLT < 150×10^9^/L |
| RPR | RDW to PLT ratio | RDW / PLT |
| S index |  | 1000 × GGT / (PLT × ALB^2^) |
